# A review of machine learning applications in soccer with an emphasis on injury risk

**DOI:** 10.5114/biolsport.2023.114283

**Published:** 2022-03-16

**Authors:** George P. Nassis, Evert Verhagen, João Brito, Pedro Figueiredo, Peter Krustrup

**Affiliations:** 1 Physical Education Department, College of Education, United Arab Emirates University, Al Ain, Abu Dhabi, United Arab Emirates; 2 Department of Sports Science and Clinical Biomechanics, SDU Sport and Health Sciences Cluster (SHSC), University of Southern Denmark, Odense, Denmark; 3 Amsterdam Collaboration on Health and Safety in Sports, Department of Public and Occupational Health, Amsterdam UMC, Amsterdam Movement Sciences, Amsterdam, Netherlands; 4 Portugal Football School, Portuguese Football Federation, Oeiras, Portugal; 5 CIDEFES, Universidade Lusófona, Lisboa, Portugal; 6 Danish Institute for Advanced Study (DIAS), University of Southern Denmark, Odense, Denmark; 7 Sport and Health Sciences, College of Life and Environmental Sciences, University of Exeter, United Kingdom

**Keywords:** Machine learning, Soccer injury risk, Data analytics, Big data, Football

## Abstract

This narrative review paper aimed to discuss the literature on machine learning applications in soccer with an emphasis on injury risk assessment. A secondary aim was to provide practical tips for the health and performance staff in soccer clubs on how machine learning can provide a competitive advantage. Performance analysis is the area with the majority of research so far. Other domains of soccer science and medicine with machine learning use are injury risk assessment, players’ workload and wellness monitoring, movement analysis, players’ career trajectory, club performance, and match attendance. Regarding injuries, which is a hot topic, machine learning does not seem to have a high predictive ability at the moment (models specificity ranged from 74.2%-97.7%. sensitivity from 15.2%-55.6% with area under the curve of 0.66–0.83). It seems, though, that machine learning can help to identify the early signs of elevated risk for a musculoskeletal injury. Future research should account for musculoskeletal injuries’ dynamic nature for machine learning to provide more meaningful results for practitioners in soccer.

## INTRODUCTION

As a branch of artificial intelligence, machine learning is being used in medicine and health sciences for some years [1, 2]. Though, machine learning is still quite new in sports science, sports medicine [3, 4] and particularly in soccer science. Machine learning use is based on the assumption that the computer and the algorithms will learn as we feed them with more data. Following data collection and data cleaning, the algorithms can build relationships among variables either without (unsupervised learning approaches) or with human assistance (supervised), who provide them with the cut-off values for specific variables. Through repeated data feeding, the computers and the algorithms will learn and will be able to identify and select, among the big number of variables, those that account for the dependent variable [3, 5, 6, 7].

One of the first studies with machine learning in soccer was published in 2014 [8]. It was focused on evaluating the technical and tactical abilities of teams during the UEFA EURO2012. Since that time, several papers have been published in soccer, focusing on machine learning and injury prediction, physical performance prediction, training load and monitoring, players’ career trajectory, club performance, and match attendance (Table 1). There are also papers on soccer match results prediction and betting, but these are not within the present review’s scope. Despite the increasing number of research papers on machine learning use in soccer, it is still unclear what machine learning does, what it can offer to soccer clubs now and in the future, and how scientists and practitioners can prepare to take advantage of machine learning capabilities. This narrative review aims to bring together information from the past to better articulate what may happen in the future regarding machine learning use in soccer, emphasizing injury risk assessment. Finally, we aim to provide practical tips for the health and performance staff in soccer clubs (i.e., physicians, physiotherapists, coaches, strength and conditioning staff, sports scientists) on how they can pave the way within the club and take competitive advantage of machine learning use. Accordingly, this manuscript is targeting the applied sports scientists and sports medicine staff who have limited knowledge and interest in the technical and computing aspects of machine learning.

**TABLE 1:** Summary of studies using machine learning approaches exclusively with soccer players as their studied population.

Study	Participants Period of study	Main outcome studied	Main finding	Topic
Ayala et al. [5]	96 male professional players, 1 season	Hamstring strain injuries predicted	The prediction model showed moderate to high accuracy	Injury risk/Injury occurrence prediction

Oliver et al. [18]	355 elite youth players aged 10–18 years old, 1 season	Injuries predicted	The best performing decision tree model provided a specificity of 74.2% and sensitivity of 55.6% with an AUC of 0.663	

Rossi et al. [17]	26 professional male players, 1 season	Non-contact injuries	Machine learning technique can detect around 80% of the injuries with about 50% precision, far better than the baselines and state-of-the-art injury risk estimation techniques	

Rommers et al. [7]	734 players, under-10 to under-15 age categories, 1 season	Non-contact injuries	Machine learning algorithm was able to identify the injured players in the hold-out test sample with 85% precision, 85% recall (sensitivity) and 85% accuracy	

Bongiovanni et al. [35]	16 male under-15 team players, 1 season	Sprint CoD, CMJ & aerobic fitness performance prediction	Anthropometric features were predictors of sprint performance and aerobic fitness, not CoD and CMJ	Physical performance prediction

Campbell et al. [29]	As the authors state "The data encompassed multiple seasons (2013–2018)and was pooled across pre-season and in-season training sessions” without including information on the data size.	Internal (sRPE) and external load (total distance covered)	Very low predictive ability	Players’ monitoring

Geurkink et al. [28]	46 elite male players, 61 training sessions, 913 observations	Predicted sRPE	sRPE was predicted accurately	

Jaspers et al. [27]	38 professional male players, 2 seasons	External and internal training load	More accurate predictions of training Rate of Perceived Exertion from external workload data in combination with pre-session wellness	

Op De Beéck et al. [26]	One soccer team (no information for duration of the study)	Future wellness items (i.e., fatigue, sleep quality, general muscle soreness, stress levels, and mood)	Wellness was predicted based on internal and external workload data. Their effect sizes indicate that the external load and internal load, separately and in combination, do not have sufficient predictive ability	

Perri et al. [36]	28 sub-elite players, 1 season	Wellness index as predicted by internal training load	Machine learning technique predicted the wellness index based on previous training day internal load	

Dick et al. [37]	Tracking data consisting of a sequence of coordinates of all players and the ball for a set of soccer games	Successful attacks	Proposed an approach to learn valuations of multiplayer positioning using positional data	Performance analysis

Goes et al. [23]	Position tracking data of 118 Dutch Eredivisie matches, containing 12424 attacks	Successful attacks	Identified dynamic formations based on position tracking data, and identified dynamic subgroups for every timeframe in a match	

Link and Hoernig [24]	Data from 60 matches in the German Bundesliga, 1 season	Models for detecting individual and team ball possession based on position data	Match event were detected automatically	

Montoliu et al. [25]	Football videos including two regular league matches played by up to four professional teams	Team activity recognition and analysis	The proposed method performed the team activity recognition task with high accuracy	

Wang [8]	Teams playing in UEFA EURO2012	Technical and tactical analysis of teams	Key performance indicators were identified	

Zago et al. [38]	13 elite female players performed a shuttle run test, wearing 6-axes inertial sensor at the pelvis level	Prediction of turn direction, speed (before/after turn) and the related positive/negative mechanical work	Good predictive ability of the machine learning algorithms	Movement analysis

Barron et al. [39]	966 outfield male players, 1 season	Identify key performance indicators that influence player’s career status	Specific technical characteristics correctly predicted 78.8% of the players’ league status with a test error of 8.3%	Player’s career trajectory

Matesanz et al. [40]	Soccer players’ transfer network among 21 European first leagues between the seasons 1996/1997 and 2015/2016	Table rank, UEFA points	Clubs with the highest transfer spending achieve better performance	Club performance

Sahin and Erol [41]	Data of 236 soccer games, 1 season	Predict the attendance demand in European soccer games	A model was proposed to predict attendance with higher accuracy	Match attendance

Note: AUC: Area Under the Curve; CoD: change of direction; CMJ: countermovement jump; sRPE: session rating of perceived exertion

### Machine learning evolution in soccer

Decision-makers (i.e., club’s owners and top managers, directors of health and performance) and team support staff/practitioners constantly strive for accurate and time-efficient methods to predict performance and assess the injury risk (or even predict the occurrence of a musculoskeletal injury). Performance prediction can help develop better training programs and shape effective game strategies, whereas injury risk prediction can protect athletes’ health and eventually optimize performance [9].

To achieve these goals, practitioners are using the latest technological advances (e.g., tracking systems such as GPS technology and inertial movement sensors, fatigue and wellness-related biological and psychological markers, screening tests) and the best analytical methods [10, 11, 12]. In the past, regression analysis has been used to assess injury risk [3] and predict sports performance. The problem with this traditional statistical analysis is that it eliminates the variables that are not linearly associated with the dependent variable. This will create a bias when searching for the interactions among variables [5, 6, 7]. The secondary problem is that traditional statistics cannot account for the effect of multiple factors on the dependent variable. For example, to assess the risk of sustaining a musculoskeletal injury, multivariate analysis models take into account the independent effect of factors in isolation, like previous injury or muscle strength, and the potential interactions between a limited number of factors (i.e., 2 to 3 each time). However, injury is a complex phenomenon [13]. Many parameters may account independently or in combination to its occurrence, including a previous injury, muscle strength imbalances, aerobic fitness or workload [14]. To account for the etiological factors’ complexity, machine learning algorithms are now being used in high-performance sports for injury prediction [7].

Machine learning analysis usually involves two phases. In phase one, an algorithm is developed based on the actual data. In phase two, this algorithm is applied to another group or a sub-group of the initial sample to access its performance [1]. A well-known tool to assess the models’ performance is the area under the curve (AUC) for the receiver operating characteristics (ROC) curve. The ROC curve is created with a true positive rate on the vertical axis and the false positive rate on the horizontal axis. The true-positive rate is known as the sensitivity, and the false-positive rate is the probability of a false alarm and is calculated as 1-specificity. The higher the AUC, the better the prediction model [5, 6, 7].

Although there is some evidence [5], we are unsure if musculo-skeletal injuries can be predicted through performance and screening tests to an accuracy suitable for valid decision making. As stated by Bahr [14], for a screening test to predict injuries, at least two things are needed: a strong relationship between the test outcome and the injury risk and the need for the test to be examined in relevant populations using appropriate tools. Both criteria have not been met with currently available tests, and it is unlikely that due to the nature of injuries, we ever will have sufficiently accurate tests available [15]. We believe these approaches (i.e., screening tests) and the statistical procedures can help identify early signs of elevated injury risk for the team supporting staff and the players to act in advance. One of the contributions of machine learning may be that it can develop the cut-off values needed from the sample data.

Despite the criticisms, the assessment of injury risk is a hot topic in soccer, as injury occurrence and the associated absence from training and match play are related to lower team ranking and lower club earnings [16]. This might be a reason explaining the growing number of research on machine learning to predict injuries [3]. If not the first study on the topic, one of the first was that by Rossi et al. [17]. The authors collected GPS data that described 26 professional male players’ workload over one season and constructed injury prediction models. The best machine learning algorithm could detect around 80% of the injuries with 50% precision and an AUC of 0.76. As the authors argued, the algorithm was “far better than the baseline and state-of-the-art injury risk estimation techniques” [17]. It should be mentioned at this point that risk estimation is not equal to risk prediction. People tend to think that predicting involves saying something will happen, whereas estimation is thought to involve how likely something is bound to happen. However, risk prediction is nothing more than calculating a probability.

Recent studies claim that injuries can be predicted by measurements taken in the pre-season. This is a challenging concept, and at least 3 studies were published in the last 2 years on the topic [5, 7, 18]. Traditionally, sports medicine and sports science staff test athletes in the pre-season for various attributes (e.g. body composition, cardiovascular fitness, muscle strength, flexibility, sprinting, and change of direction ability) to analyze the data and assess the injury risk at specific time points in the course of the season [5, 6, 7, 18]. The assumption is that any disadvantage diagnosed with these tests will result in an elevated risk of musculoskeletal injury. Machine learning algorithms have shown to predict injuries with moderate to high accuracy (Table 1). Despite that, we are unsure of what this “moderate to high accuracy” means in clinical terms.

Ayala et al. [5] screened 96 players regarding their history of injuries, psychological and neuromuscular risk factors as part of their pre-season assessment. The best model showed moderate to high accuracy with an AUC score of 0.83, a true positives rate of 77.8% and true negatives of 83.8% in predicting hamstring strain injuries. Similarly, Oliver et al. [18] performed neuromuscular testing in pre-season and followed 355 youth soccer players for the entire soccer season. The machine learning model showed a specificity of 74.2% and sensitivity of 55.6%, with an AUC of 0.66. Interestingly, logistic regression provided a specificity of 97.7% and a sensitivity of 15.2%, with an AUC curve of 0.66, suggesting a much higher sensitivity with machine learning in predicting injuries than traditional statistics [18. However, it must be noted that an AUC of 0.66 is not acceptable for an injury prediction tool [18]. Rommers et al. [7] tested 734 young players for strength, flexibility, agility, and endurance in pre-season. Anthropometric data and occurring injuries were also recorded throughout the entire season. The machine learning algorithm predicted injury with a precision of 86% in the training data set [7].

In conclusion, it seems that machine learning techniques can assess the injury risk with moderate to high accuracy based on pre-season screening data. Despite that, we are still unsure what this “moderate to high accuracy” means in clinical terms. Furthermore, it should be mentioned that injury risk estimation is not equally interpreted as injury prediction. Even though fundamentally, in prediction models, one looks for the probability of an outcome, it is easily (and most frequently) wrongly interpreted as the estimation and outcome ‘will’ occur, instead of ‘can’ occur.

### Challenges with regards to injury prediction

The problem with the approaches mentioned above is that data collected months before an injury cannot account for the dynamic nature of soccer activities and the changes that may happen to the players. Athletes are exposed to a specific workload that may modify the relation between a parameter and the injury risk [14]. Indeed, it is assumed that the accuracy with machine learning can be improved with data (e.g., workload, readiness, physiological and contextual data like training ground condition, shoe type) collected as close to the event (the injury occurrence) possible. Real-time data on athlete’s physiology (e.g., heart rate, body temperature), mechanical responses (e.g., fatigue-induced alterations in mechanics of movements), and other contextual factors (e.g., pitch condition, shoe-surface properties) could add value and improve the accuracy of machine learning techniques in the future. This will be one of the biggest challenges soon with integrating big data and machine learning applications in a way that adds a competitive advantage in sports.

There are at least two more problems associated with the use of machine learning in soccer injury prediction: 1) the low incidence of specific injuries that prevent algorithms from reaching a higher accuracy [19]; and 2) the uncertainty when applying machine learning algorithms to another setting or using slightly different data collection procedure than the original one. Regarding the first point, in elite soccer, one can expect about 50 injuries per team during a season, a very small number to create prediction models with high accuracy in a single team [19]. The situation becomes more challenging when considering the different types of injuries within a soccer squad. The problem of low cases in most studies is called the im-balanced data-sets problem in data science [5, 6, 7, 20], because one class (in this case, the injured athletes) is underrepresented in the data-set [20].

Machine learning models are usually biased towards the majority class (in this case, the non-injured athletes), which means the algorithms may inaccurately predict the minority class (the injury cases). Experts in the field have suggested technical solutions to this problem, and the readers may find the technical details elsewhere [5, 6, 7, 20]. However, another potential solution is to collect data from many teams and treat them as one sample. As an example, all teams of a national league could send their data to a common database. Nevertheless, what works in one team may not work in another. As stated before, injury risk is context-specific and essential factors like the team, medical support, and staff communication are crucial elements [21]. Therefore, we need more data specific to the players. Then, we move to the second challenge, which is the uncertainty when applying a machine-learning algorithm to a different setting [5, 6, 7]. It seems that even a slight deviation in the procedure of data collection may affect the outcome. As suggested, if a new test is added or an existing one is executed differently from the original one used for the algorithm development, this could affect the algorithm performance [5, 6, 7].

### What can soccer learn from other sports and scientific fields?

Besides injuries, the occurrence of illnesses is of major concern in sports due to potential loss of training and hence the risk of under-performance. An earlier study has developed predictive models for illnesses based on workload data in rugby league players, and this is an interesting dimension with high practical importance [22]. Thirty-two professional rugby league players were recruited, their internal training and match load were recorded, and perceptual well-being ratings were collected for 29 weeks during a rugby league season. A decision-tree model was developed with the risk factors contributing to the self-reported illness and their cut-off values [22]. Overall, a reduction in well-being combined with an increased internal training load were the main contributors to the self-reported illness occurrence. The area under the ROC curve ranged from 46% to 80%, depending on the model [22]. The ROC range may indicate that more work is needed for the machine learning algorithms to provide risk assessments with higher accuracy based on this nature of data.

### Machine learning use in other outcome measures in soccer

In soccer, performance analysis is the topic with most studies related to machine learning published so far (Table 1). Most of these studies are related to the automatic detection of match events [23, 24, 25]. The development of algorithms based on machine learning, which will detect match events with higher accuracy faster, is advantageous for high-performance teams who analyze big data (e.g., data of their team and those of the opponents) [24].

An interesting dimension is applying machine learning for predicting a player’s wellness [26]. The authors used machine learning algorithms to predict wellness based on external and internal load data during training sessions. Wellness was calculated every morning from scores on perceived fatigue, sleep quality, general muscle soreness, stress levels, and mood state. Machine learning was applied to develop predictive models for the next day’s wellness score based on the last day’s training load (e.g., external and internal load) and wellness score. Although this is a promising dimension, the analysis showed that the data did not have sufficient predictive ability for the next day’s wellness score [26]. In the same topic, Jasper et al. [27] and Geurkink et al. [28] have employed machine learning to predict training session’s ratings of perceived exertion (sRPE) from external workload data (i.e., GPS-derived). The analyses showed that sRPE could be predicted accurately for sRPE values below 8 [28]. This work is very relevant because coaches may design better training methods using past GPS-derived data to predict sRPE and hence the internal workload of the players with higher accuracy.

If wellness data have a predictive value for subsequent training, practitioners would inform coaches to modify training volume and intensity when necessary. This was the aim of a recent study conducted by Campbell et al. [29], who analysed a pool of data captured and stored in an athlete’s management system platform. As the authors state, “the data encompassed multiple seasons (2013–2018) and was pooled across pre-season and in-season training sessions” without including information on the data size. This study showed the very low predictive ability of wellness data on internal (s-RPE) and external load (total distance covered) in soccer players [29].

### Ethical and technological considerations with the use of machine learning

As with every new technology, there are some ethical considerations regarding the unintended use of machine learning [30]. It is tempting to use existing data that was not directly gathered for machine learning studies. Consent, as such, is not always given for the use of data by the athletes. Practitioners should carefully review the process and ensure their actions obey the law and follow the authorities and organizational policies. For instance, the use of databases containing personal information collected without the consent of the individual(s) is a major issue, and practitioners should stick to the rules. This is extremely important regarding the use of medical records that contain sensitive personal data [31]. Therefore, data protection experts’ contribution and approval are highly recommended when using machine learning in soccer.

Another consideration is the use of machine learning for decision-making. This brings management and leadership aspects to the front stage of successful management of risk using machine learning [32]. For instance, how does the supporting team staff deal with false-positive cases communicated to the coach or the true-positives not communicated? What is the acceptable threshold for the identified high-risk cases to be communicated to the coaches and the top management? Education of ev eryone involved with data analysis and decision-making is therefore of utmost importance [33].

In the future, the predictive models could integrate data related both to intrinsic factors (i.e. demographics, anatomical, physiological, psychological profile) and extrinsic factors (i.e. workload and environmental data). The addition of veracity metrics related to physical testing and workload monitoring could also help. A recent paper reported that data veracity (accuracy, reliability, and quality of data) was found for 54% of tools and 23% of the parameters used in testing and workload monitoring in soccer studies [34].

## CONCLUSIONS

In this narrative review paper, we have attempted to shed some light on the use of machine learning in soccer, emphasizing injuries, given practitioners’ interest on this topic. Machine learning does not seem to have high predictive ability in every setting. It seems that machine learning can help to identify early signs of elevated risk for a musculoskeletal injury. Future research should account for musculoskeletal injuries’ dynamic nature for machine learning to provide more meaningful results. Performance analysis is the area with most research at the moment. Knowledge in this area is growing and is having practical applications in the club setting.

## Conflict of interest

The authors declare that they have no competing interests.
